# Psychometric properties and moderated mediation analysis of the ICIQ-NQOL in Chinese primary care patients with nocturia

**DOI:** 10.1186/s41687-024-00756-2

**Published:** 2024-08-07

**Authors:** Edmond Pui Hang Choi, Chanchan Wu, Lily Man Lee Chan, Heidi Sze Lok Fan, Jojo Yan Yan Kwok, Pui Hing Chau, Esther Yee Tak Yu, Samuel Yeung Shan Wong, Cindy Lo Kuen Lam

**Affiliations:** 1 School of Nursing, Li Ka Shing Faculty of Medicine, The University of Hong Kong, 5/F, Academic Building, 3 Sassoon Road Pokfulam, Hong Kong SAR, China; 2 School of Nursing, Faculty of Health and Social Development, University of British Columbia, Kelowna, Canada; 3 Centre on Behavioral Health, Faculty of Social Sciences, The University of Hong Kong, Hong Kong SAR, China; 4 Department of Family Medicine and Primary Care, School of Clinical Medicine, Li Ka Shing Faculty of Medicine, The University of Hong Kong, Hong Kong SAR, China; 5 The Jockey Club School of Public Health and Primary Care, The Chinese University of Hong Kong, Hong Kong SAR, China; 6 Department of Family Medicine, The University of Hong Kong Shenzhen Hospital, Shenzhen, China

**Keywords:** Nocturia, Quality of life, Psychometric evaluation, Patient-reported outcomes, Moderated mediation, Validation, Chinese

## Abstract

**Background:**

Many individuals consider nocturia a significant nuisance, leading to a reduced health-related quality of life (HRQOL). However, there has been a lack of psychometrically sound patient-reported outcome measures to assess the impact of nocturia on patients in Chinese contexts. This study aimed to translate, culturally adapt, and validate the International Consultation on Incontinence Questionnaire Nocturia Quality of Life Module (ICIQ-NQOL) for use among primary care patients in Hong Kong, China. Additionally, it sought to investigate the mechanisms that link nocturia and sleep quality with HRQOL by employing moderated mediation analysis.

**Methods:**

The traditional Chinese version of the ICIQ-NQOL was developed through iterative translations, cognitive debriefing interviews, and panel reviews. The psychometric evaluation included assessments of factor structure, convergent validity, concurrent validity, known-group validity, internal consistency, test-retest reliability and responsiveness. Study instruments included the ICIQ-NQOL, International Prostate Symptom Score (IPSS), Pittsburgh Sleep Quality Index (PSQI), and a modified Incontinence Impact Questionnaire-Short Form (IIQ-7).

**Results:**

A total of 419 primary care patients were recruited from general outpatient clinics, among whom 228 experiencing an average of two or more nocturia episodes per night over the past four weeks. Confirmatory factor analysis supported the two-factor structure of the ICIQ-NQOL. Concurrent validity was confirmed by moderate correlations between the IIQ-7 total score and the total score as well as two domain scores of the ICIQ-NQOL (*r* ranging from 0.43 to 0.49, all *p* < 0.001). The ICIQ-NQOL also had moderate correlations with the IPSS total symptom score (*r* ranging from 0.40 to 0.48, all *p* < 0.001). Convergent validity was supported by moderate correlations between the global PSQI score and the total score as well as two domain scores of the ICIQ-NQOL (*r* ranging from 0.42 to 0.52, all *p* < 0.001). Known-group comparisons showed that the ICIQ-NQOL could differentiate between patients with and without nocturia in terms of sleep/energy domain score (*p* < 0.001), bother/concern domain score (*p* < 0.001), and total score (*p* < 0.001), each demonstrating a moderate Cohen’s *d* effect size. Item-total correlations corrected for overlap exceeded 0.4, and Cronbach’s alpha coefficients were greater than 0.7. Test-retest reliability was confirmed with intraclass correlation coefficients exceeding 0.7 among patients reporting no change in their nocturia symptoms at a 2-week follow-up. Regarding responsiveness, the Cohen’s *d* effect sizes for differences in domain and total scores between the baseline and 2-week follow-up assessments were greater than 0.3 among patients showing improvement in nocturia. Our moderated mediation analysis indicated that sleep quality significantly moderated the impact of nocturia on HRQOL, with a notably stronger indirect effect among females compared to males.

**Conclusions:**

The ICIQ-NQOL is a reliable and valid instrument for assessing the HRQOL in primary care patients suffering from nocturia. The findings advocate for gender-specific approaches in the management and treatment of nocturia to optimize HRQOL.

## Background

The International Continence Society defines nocturia as the need to awaken and void urine during the main sleep period [1]. Recognized as one of the most common lower urinary tract symptoms (LUTS), the prevalence of nocturia is substantial among different age groups and nationalities. The EpiLUTS study, encompassing populations in the United States (US), United Kingdom, and Sweden, has found that a majority of individuals over the age of 40—specifically, 69% of men and 76% of women—experience at least one episode of nocturia each night [2]. Furthermore, approximately one-third of this demographic faces two or more nocturnal awakenings to urinate, with 28% of men and 34% of women affected [2]. Parallel findings in East Asia, including mainland China, Taiwan, and South Korea, reveal a similar pattern, with a reported 36% of those aged 40 and above experiencing two or more nocturia episodes nightly [3]. These data collectively reinforce the notion that nocturia is a globally prevalent health concern.

The aetiology of nocturia is multifaceted [4], encompassing urological as well as non-urological elements. Urological conditions, including overactive bladder syndrome and benign prostatic hyperplasia, are frequently associated with nocturia. However, the role of non-urological factors is also considerable. Comorbidities such as arthritis and cardiovascular diseases have been linked to nocturia, as highlighted by findings from the EpiLUTS study [2]. Additionally, medications used to treat these chronic conditions, along with the physiological alterations they induce, might worsen nocturia symptoms [2].

The effects of nocturia are diverse and significant. Numerous studies indicate that individuals often find nocturia to be a considerable nuisance, which can result in a diminished health-related quality of life (HRQOL) [5]. Research has indicated a correlation between the severity of nocturia and its negative impact on HRQOL and overall discomfort. For instance, a study in Taiwan revealed that individuals experiencing more episodes of nocturia per night tend to have poorer HRQOL [6]. Research conducted within a community setting in the US has demonstrated that nocturia has a detrimental effect on HRQOL, as measured by the Short Form-36 Health Survey (SF-36) [7]. Similarly, a Swedish study indicated that nocturia patients experienced a significant decrease in the vitality domain as measured by the SF-36, with an average decline of 14 points on the SF-36 scale [8]. This pronounced drop in vitality for nocturia sufferers is concerning, especially since a decrease of 10 points in vitality has been linked to a heightened risk of negative health outcomes. These include an increased chance of being unable to work due to health issues (odds ratio [OR] of 1.62), the probability of job loss within a year (OR of 1.28), a greater likelihood of hospitalization within a year (OR of 1.17), and a higher risk of death within 18 months (hazard ratio ranging from 1.21 to 2.39) [9]. Similarly, individuals with nocturia often report disrupted sleep. In a US-based study, over half of the participants aged between 55 and 84 years identified nocturia as a frequent cause of their sleep disruption, occurring every night or almost every night [10]. The same study highlighted nocturia as a significant risk factor for insomnia (OR of 1.75) and diminished sleep quality (OR of 1.71) [10]. Such sleep disturbances can have a profound effect on daily functioning and overall HRQOL. A systematic review revealed that the impact of nocturia on work productivity is considerable, paralleling the effects seen with other prevalent chronic conditions like depression and chronic obstructive pulmonary disease [11]. In Hong Kong, men seeking urological care for LUTS most commonly reported nocturia, and it was also among the symptoms they found most troubling. When compared to individuals from other regions, those from Hong Kong appeared to experience more pronounced symptoms and a higher degree of bother. Despite this, they were less likely to have undergone treatment and tended to express lower satisfaction with the treatments they had received [12].

Patient-reported outcome instruments offer a standardized approach to quantifying the effects of nocturia on patients. Various questionnaires have been tailor-made to assess the adverse consequences of different urinary symptoms within specific populations. For instance, the International Prostate Symptoms Score (IPSS) is designed for men with benign prostatic hyperplasia (BPH) [13], while the Incontinence Quality of Life Questionnaire targets those suffering from urinary incontinence. Due to the distinctive nature and consequences of different urinary symptoms, instruments meant for conditions like incontinence may not be appropriate for evaluating the impact of nocturia [14]. Moreover, while the single HRQOL item included in the IPSS is frequently utilized to gauge the influence of LUTS on HRQOL, its generality may render it insufficiently sensitive to fully capture the specific effects of nocturia on HRQOL [15].

The International Consultation on Incontinence Questionnaire Nocturia Quality of Life Module (ICIQ-NQOL) was developed specifically for individuals experiencing nocturia [14, 16]. Developed by Abraham et al. in 2004, it was the first questionnaire developed to assess nocturia’s impact on HRQOL [14], with a particular emphasis on the effects of nocturia on the patient’s daily living. It can be used as an outcome measure to evaluate the burden of nocturia and to assess the impacts of different treatment modalities [17]. The questionnaire has been translated into several languages, including Danish, Italian, Korean, and Thai [16, 17]. However, the applicability and psychometric properties of this questionnaire within the Hong Kong Chinese population were unclear, which hindered its implementation in this group. Therefore, developing a Chinese version of the ICIQ-NQOL for use in the Hong Kong Chinese population was important to enhance the assessment of the impact of nocturia and the outcomes of interventions and healthcare services for treating patients with nocturia.

The study had two primary objectives: (i) to translate and culturally adapt the ICIQ-NQOL for use with primary care patients in Hong Kong who experience nocturia, and evaluate its psychometric properties; and (ii) to identify factors associated with the impact of nocturia on HRQOL among this population.

## Methods

### Study design

The study comprised two phases. The first phase focused on the translation and cross-cultural adaptation of the ICIQ-NQOL to ensure relevance and comprehension in the context of Chinese culture. The second phase entailed an observational study, which included a comprehensive psychometric evaluation of the newly translated traditional Chinese version of the ICIQ-NQOL to assess its validity and reliability, as well as to explore factors associated with nocturia-specific HRQOL.

### Phase 1: translation and cross-cultural adaptation of the ICIQ- NQOL

The translation and cultural adaptation of the ICIQ-NQOL into traditional Chinese adhered to the Principles of Good Practice for the Translation and Cultural Adaptation Process for Patient-Reported Outcome (PRO) Measures as outlined by the Professional Society for Health Economics and Outcomes Research [18]. The steps of the translation process were as follows:

1. **Preparation**: The necessary authorization to utilize the instrument was obtained from the original developer.
2. **Forward Translation**: Two separate translators independently rendered the original English version into traditional Chinese, with the work commissioned to a professional translation service located in Hong Kong.
3. **Reconciliation**: A registered nurse, who is a native speaker of Chinese, amalgamated the two independent forward translations into a single, unified Chinese version. The first author of the paper then conducted a review of this harmonized version for conceptual alignment.
4. **Back Translation**: A translator, blinded to the original English document, back-translated the harmonized Chinese version into English. This was also conducted by a professional service in Hong Kong.
5. **Review of Back Translation**: The original developer reviewed the back-translated English version to ensure that it accurately reflected the content of the original instrument.

Cognitive debriefing interviews were conducted to evaluate the content validity of the traditional Chinese version of the ICIQ-NQOL. Three aspects were assessed: the instrument’s clarity, relevance, and appropriateness [19].

A convenience sample of 10 Chinese patients with nocturia, consisting of five males and five females, was recruited from the general outpatient clinics of the Hospital Authority in the Hong Kong West Cluster based on the inclusion criteria: (i) aged 18 or older; (ii) having an average number of nocturia episodes of two or more per night in the past four weeks; and (iii) able to understand Chinese.

Each patient completed the traditional Chinese version of the ICIQ-NQOL, followed by a semi-structured interview that included five questions about their perception of the instrument. The first three questions required patients to rate each item in the ICIQ-NQOL on a four-point Likert scale in terms of clarity, relevance, and appropriateness. Ratings ranged from 1 (not at all clear/relevant/appropriate) to 4 (completely clear/relevant/appropriate). The final two questions asked patients to describe their understanding of each item and to suggest any rewording for parts they found unclear, irrelevant, or inappropriate [19].

The research team members reviewed the findings from the cognitive debriefing interviews, with the review identifying any discrepancies between the patients’ interpretations and the intended meaning of the translated ICIQ-NQOL. Patient-recommended rewordings were considered, and question items were revised as necessary to enhance clarity, relevance, and appropriateness.

Input from healthcare professionals was also sought to ensure content validity. A panel of six healthcare professionals, comprising three nurses and three physicians, reviewed each translated item of the ICIQ-NQOL for clarity, relevance, and appropriateness. Further revisions to the questionnaire items were made where necessary.

### Phase 2: psychometric evaluation and exploring factors associated with HRQL

#### Sampling, patient recruitment, and procedure

The study was conducted at the general outpatient clinics of the Hong Kong West Cluster. Patients were recruited using convenience sampling. The inclusion criteria included: (i) being aged 18 or above, and (ii) having the ability to understand and read Chinese. Patients were excluded if they were too ill to give consent or complete the questionnaire.

Participants were divided into two groups based on their nocturia status: those with nocturia and those without. In alignment with previous research findings [3], nocturia was defined as experiencing two or more episodes per night. The threshold of two voiding episodes per night was chosen for two main reasons: (i) the definition of nocturia by the International Continence Society as at least one episode per night could result in a very high reported prevalence [3], and (ii) a cutoff of two or more episodes per night is considered more clinically significant [20, 21]. Conversely, participants reporting fewer than two episodes of nocturia per night were classified as not having nocturia.

A subset of patients experiencing an average of two or more nocturia episodes per night over the previous four weeks was invited for a follow-up assessment after two weeks to assess the test-retest reliability.

#### Study instruments

##### Nocturia

To evaluate nocturia, participants were asked to report the average number of nocturia episodes they experienced per night over the previous four weeks. Their responses were recorded using a five-point Likert scale with options of 0, 1, 2, 3, or 4 or more episodes per night [3].

##### International Consultation on Incontinence Questionnaire Nocturia Quality of Life Module (ICIQ-NQOL)

The ICIQ-NQOL is a questionnaire consisting of 13 items designed to assess the impact of nocturia on individuals’ HRQOL, with a recall period of four weeks. Responses to the items are given on a 5-point Likert scale, with scores ranging from 0 to 4. The total score, as outlined by the scoring method of the ICIQ-NQOL, is the sum of all individual item scores, which can range from 0 to 58. A higher total score indicates a greater negative impact on HRQOL. Based on the research by Abraham et al., the questionnaire yields scores for two domains: the sleep/energy domain (compromising items 1 to 5 and 7) and the bother/concern domain (comprising items 6 and 8 to 12). The global quality of life item (item 13) is not included in either domain score [16].

##### Pittsburgh Sleep Quality Index (PSQI)

The Chinese version of the PSQI, a self-report questionnaire, was utilized to measure sleep quality. It is widely applied in both clinical practice and research and has demonstrated strong psychometric properties. The PSQI covers a recall period of one month and comprises 19 items, yielding seven component scores that reflect different facets of sleep quality: subjective sleep quality, sleep latency, sleep duration, habitual sleep efficiency, sleep disturbances, use of sleeping medication, and daytime dysfunction. As per the original developers of the scale [22], each component is scored on a scale from 0 to 3. For instance, in the sleep duration component, a duration of more than 7 h scores 0, while sleeping less than 5 h scores 3. Similarly, for the subjective sleep quality component, the scores are distributed as follows: ‘very good’ scores 0, ‘fairly good’ scores 1, ‘fairly bad’ scores 2, and ‘very bad’ scores 3. These scores from all seven components are combined to produce a global score ranging from 0 to 21, where a higher global score indicates poorer sleep quality [23].

##### Modified incontinence impact questionnaire-short form (IIQ-7)

The modified IIQ-7 includes seven questions designed to evaluate the impact of LUTS on HRQOL. Each item is answered using a 4-point Likert scale format ranging from 0 to 3. Scores range from 0 to 21, with higher scores indicating poorer LUTS-specific HRQOL. This version of the IIQ-7 has been validated in the Hong Kong Chinese population, with its 2-week test-retest reliability, internal consistency, known-group validity, and convergent validity all established [24].

##### International prostate symptoms score (IPSS)

The IPSS comprises seven items that measure the severity of lower urinary tract symptoms, specifically: incomplete bladder emptying, frequency of urination, intermittency, urgency, weak urine stream, straining, and nocturia. The total symptom score is calculated by summing the responses to these seven questions. In addition, there is a single item that assesses the impact of LUTS on HRQOL. Higher scores on the IPSS indicate more severe symptoms or poorer HRQOL. The IPSS has been validated for use in Hong Kong with both male and female primary care patients [25, 26].

##### Global rating of Change (GRC) Scale

The single-item GRC scale was used to ask patients to rate the change in their nocturia symptoms since the initial baseline assessment. They were asked to respond using a 7-point Likert scale, with the following options: 1 = much worse, 2 = worse, 3 = minimally worse, 4 = no change, 5 = minimally improved, 6 = improved, and 7 = much improved. The GRC scale was administered exclusively at the two-week follow-up assessment [25].

Basic socio-demographic data such as age and sex were collected using a structured questionnaire.

### Analysis plan

For the Phase 1 translation and cross-cultural adaptation of the ICIQ-NQOL, content validity indices (CVI) for each item’s clarity, relevance, and appropriateness were determined by the proportion of subjects who gave the item a rating of 3 or 4 on a four-point scale. The standard for good content validity was set as a CVI of 80% or above [19].

For psychometric evaluation, the following analyses were conducted:

- **Factor Structure**: Confirmatory factor analysis (CFA) was conducted to examine the ICIQ-NQOL’s factor structure as proposed by Abraham et al. [16]. The model’s goodness-of-fit statistics were evaluated using standardized root mean square residual (SRMR), root mean square error of approximation (RMSEA), comparative fit index (CFI), and Tucker-Lewis index (TLI), as recommended by Hu and Bentler. A model fit was considered good if the SRMR was close to or below 0.08, RMSEA was close to or below 0.06, and CFI and TLI values were greater than 0.9—with values above 0.90 being acceptable and above 0.95 being excellent [27].
- **Concurrent Validity**: The correlation between the ICIQ-NQOL, IPSS and the modified IIQ-7 was assessed, as they are urinary problem-specific instruments and are theoretically related. Pearson’s correlation coefficients were computed to measure the strength of the relationship between the ICIQ-NQOL’s scores, IPSS scores and the modified IIQ-7’s total score.
- **Convergent Validity**: The association between the ICIQ-NQOL and the PSQI was investigated. The theoretical basis for this analysis is the hypothesis that patients with nocturia would experience poorer sleep quality, which in turn affects HRQOL. The correlations between the ICIQ-NQOL’s scores and the PSQI’s compositions and global scores were examined.
- **Known-Group Validity**: Comparisons were made between primary care patients with and without nocturia to assess known-group validity. It was hypothesized that patients with nocturia would have higher ICIQ-NQOL scores, indicating poorer HRQOL. An independent *t*-test was used to compare the ICIQ-NQOL scores between patients with nocturia and those without. Cohen’s *d* effect size was also calculated.
- **Internal Consistency**: To evaluate internal consistency, Cronbach’s alpha and item-to-total correlation coefficients were calculated. A Cronbach’s alpha of 0.7 or higher was indicative of acceptable internal consistency, while a corrected item-to-total correlation coefficient ≥ 0.4 was considered adequate [25].
- **Test-Retest Reliability**: Patients with nocturia were invited to complete the ICIQ-NQOL two weeks after their initial baseline assessment to assess the test-retest reliability of the instrument over that period. The analysis employed a two-way mixed-effects model with a single measurement and absolute agreement criterion [28]. An intraclass correlation coefficient (ICC) of 0.7 or above was considered to indicate good test-retest reliability [25].
- **Responsiveness**: To evaluate the internal responsiveness of the ICIQ-NQOL to changes, paired *t*-tests were used to compare the mean change in ICIQ-NQOL scores between baseline and the two-week follow-up assessment among patients who reported changes in their nocturia symptoms as measured by the GRC scale [29, 30]. Additionally, Cohen’s *d* effect size was calculated to assess the magnitude of these changes. The values of effect size were interpreted as trivial (< 0.2), small (≥ 0.2 and < 0.5), moderate (≥ 0.5 and < 0.8) and large (≥ 0.8). Internal responsiveness was supported if the difference is interpreted as small or the above [31].

Linear regression analysis was employed to investigate factors associated with ICIQ-NQOL scores. Building on the results of the linear regression, we further examined the interrelationships among nocturia, sleep quality, and HRQOL. To assess potential moderated mediation effects—also known as conditional indirect effects—Hayes’s Process Model 7 was applied [32]. In this analysis, nocturia status functioned as the independent variable and the global PSQI score acted as the mediator. The ICIQ-NQOL scores were the dependent variables of interest, with sex serving as the moderating variable. All data analyses were conducted using Statistical Package for Social Sciences (SPSS) (version 28.0.1.0) and Jeffrey’s Amazing Statistics Program (JASP) (version 0.16.3), and a *p*-value of < 0.05 was considered statistically significant.

### Sample size

The sample size calculation was based on the following justifications. First, considering that for the CFA of the population model, at least 300 participants are recommended [33]. Second, according to the sample size calculation [34], a minimum of 404 participants is required to achieve a CFI of 0.95. This calculation is based on a model consisting of 12 items and 2 factors, with average factor loadings of 0.5 and an average factor correlation of 0.9. This sample size ensures an 80% statistical power and adheres to a 5% significance level. Third, based on findings from a prior study [35], the Cohen’s d effect size for differences in the physical component of HRQOL, as measured by the 12-Item Short Form Health Survey version 2, was 0.38. This effect size was observed between patients experiencing nocturia (≥ 2 episodes per night) and those without (< 2 episodes per night). To detect this effect size with an independent *t*-test, ensuring 80% statistical power and a 5% significance level, we required at least 110 patients in each group, totalling at least 220 patients. Third, for the assessment of test-retest reliability, a minimum of 50 patients was required [36].

## Results

### Content validity

A convenience sample of 10 Chinese patients with nocturia, five males and five females, with a mean age of 68 years (standard deviation *[SD]* of 6.3), was recruited. A panel of six healthcare professionals, including three nurses and three doctors, was also consulted to ensure the content validity. The CVI scores for clarity, relevance, and appropriateness reached 100% for nearly all items. Exceptions included items related to physical activities, where CVI scores for relevance and appropriateness were 83% among healthcare professionals. Similarly, items concerning fluid restriction and preoccupation with waking at night had clarity scores of 83%. Following cognitive debriefing, the research team refined the wording of the items pertaining to fluid restriction and preoccupation with waking at night to enhance clarity further.

### Study participants

A total of 419 primary care patients were recruited from the general outpatient clinics within the Hong Kong West Cluster from August 2022 to September 2023. Of these, 228 patients (54.4%) reported experiencing an average of two or more nocturia episodes per night over the past four weeks. Compared to patients without nocturia, those experiencing nocturia were typically older, with a mean age of 66.8 versus 61.4 years (*p* < 0.001). Additionally, a higher percentage of patients with nocturia were male (70.2% vs. 65.4%, *p* = 0.031), not employed (70.2% vs. 48.7%, *p* < 0.001), had lower monthly household incomes (70.6% vs. 43.5%, *p* < 0.001), and had lower educational attainment (74.1% vs. 60.2%, *p* < 0.001). Table 1 shows the patient characteristics.

**Table 1:** Characteristics of study participants

	Overall (*n* = 419)	Nocturia < 2 episodes per night (*n* = 191)	Nocturia ≥ 2 episodes per night (*n* = 228)	*p*-value^a^
**Mean age (SD)**	64.3 (8.9)	61.4 (8.6)	66.8 (8.3)	< 0.001
**Sex**,** n (%)**				0.031
Male	285 (68.0)	125 (65.4)	160 (70.2)	
Female	134 (32.0)	66(34.6)	68 (29.8)	
**Marital status**,** n (%)**				0.940
Married	330 (78.8)	149 (78.0)	181 (79.4)	
Not married	87 (20.8)	41 (21.5)	46 (20.2)	
Refused to answer	2 (0.5)	1 (0.5)	1 (0.4)	
**Employment status**,** n (%)**				< 0.001
Employed	163 (38.9)	96 (50.3)	67 (29.4)	
Not employed	253 (60.4)	93 (48.7)	160 (70.2)	
Refused to answer	3 (0.7)	2 (1.0)	1 (0.4)	
**Monthly household income**,** n (%)**				< 0.001
HKD 20,000 or above	105 (25.1)	65 (34.0)	40 (17.5)	
Less than HKD 20,000	244 (58.2)	83 (43.5)	161 (70.6)	
Refused to answer	70 (16.7)	43 (22.5)	27 (11.8)	
**Educational level**,** n (%)**				< 0.001
Tertiary education	129 (30.8)	75 (39.3)	54 (23.7)	
Primary/Secondary education	284 (67.8)	115 (60.2)	169 (74.1)	
Refused to answer	6 (1.4)	1 (0.5)	5 (2.2)	

*Note *^a^An independent *t*-test was used for continuous variables, and chi-square tests were used for categorical variables

*Abbreviations HKD* Hong Kong Dollar, *SD* standard deviation

### Validity

Regarding the factor structure of the ICIQ-NQOL, the CFA supported the two-factor structure for the ICIQ-NQOL as proposed by Abraham et al. [16] The RMSEA was 0.058, and the SRMR was 0.062. The CFI stood at 0.99, with a TLI of 0.99.

Concerning concurrent validity, the IIQ-7 total score demonstrated a moderate correlation with the ICIQ-NQOL’s sleep/energy domain score (*r* = 0.46, *p* < 0.001), bother/concern domain score (*r* = 0.43, *p* < 0.001), and the overall total score (*r* = 0.49, *p* < 0.001). The IPSS total symptom score also demonstrated a moderate correlation with the ICIQ-NQOL’s sleep/energy domain score (*r* = 0.43, *p* < 0.001), bother/concern domain score (*r* = 0.40, *p* < 0.001), and the overall total score (*r* = 0.48, *p* < 0.001). In terms of convergent validity, the global PSQI score also exhibited a moderate correlation with the ICIQ-NQOL’s sleep/energy domain score (*r* = 0.52, *p* < 0.001), bother/concern domain score (*r* = 0.42, *p* < 0.001), and the total score (*r* = 0.51, *p* < 0.001). Table 2 shows the results of concurrent and convergent validity.

**Table 2:** Concurrent and convergent validity

	ICIQ-NQOL					
	Sleep/energy domain		Bother/concern domain		Total score	
	*r* (95% CI)	*p*-value	*r* (95% CI)	*p*-value	*r* (95% CI)	*p*-value
**IIQ-7 total score** (*n* = 419)	0.46 (0.39, 0.54)	< 0.001	0.43 (0.35, 0.51)	< 0.001	0.49 (0.42, 0.56)	< 0.001
**IPSS scores**						
Total symptom score (*n* = 419)	0.43 (0.35, 0.51)	< 0.001	0.40 (0.32, 0.48)	< 0.001	0.48 (0.40, 0.55)	< 0.001
Quality of life score (*n* = 419)	0.34 (0.25, 0.42)	< 0.001	0.42 (0.34, 0.50)		0.44 (0.36, 0.51)	< 0.001
**PSQI scores**						
Component 1: Subjective sleep quality (*n* = 415)	0.45 (0.37, 0.52)	< 0.001	0.38 (0.30, 0.46)	< 0.001	0.44 (0.36, 0.52)	< 0.001
Component 2: Sleep latency (*n* = 417)	0.27 (0.17, 0.35)	< 0.001	0.18 (0.08, 0.27)	< 0.001	0.25 (0.16, 0.34)	< 0.001
Component 3: Sleep duration (*n* = 414)	0.22 (0.13, 0.31)	< 0.001	0.13 (0.04, 0.23)	0.007	0.19 (0.10, 0.28)	< 0.001
Component 4: Sleep efficiency (*n* = 413)	0.31 (0.22, 0.40)	< 0.001	0.28 (0.19, 0.36)	< 0.001	0.33 (0.24, 0.41)	< 0.001
Component 5: Sleep disturbance (*n* = 417)	0.32 (0.23, 0.40)	< 0.001	0.32 (0.23, 0.40)	< 0.001	0.35 (0.27, 0.43)	< 0.001
Component 6: Use of sleep medication (*n* = 417)	0.18 (0.09, 0.27)	< 0.001	0.12 (0.03, 0.21)	0.014	0.17 (0.08, 0.26)	< 0.001
Component 7: Daytime dysfunction (*n* = 417)	0.45 (0.37, 0.53)	< 0.001	0.36 (0.28, 0.44)	< 0.001	0.43 (0.35, 0.50)	< 0.001
Global PSQI score (*n* = 411)	0.52 (0.45, 0.59)	< 0.001	0.42 (0.33, 0.49)	< 0.001	0.51 (0.44, 0.58)	< 0.001

*Note *Pearson’s correlation coefficients were computed

*Abbreviations CI* confidence interval, *ICIQ-NQOL* International Consultation on Incontinence Questionnaire Nocturia Quality of Life Module, *IIQ-7* Modified Incontinence Impact Questionnaire-Short Form, *IPSS* International Prostate Symptoms Score, *PSQI* Pittsburgh Sleep Quality Index

In the known-group comparison, the ICIQ-NQOL was able to differentiate between patients with nocturia and those without in terms of the sleep/energy domain score (*p* < 0.001), bother/concern domain score (*p* < 0.001), and the total score (*p* < 0.001), each demonstrating a moderate Cohen’s *d* effect size. Table 3 shows the results of the known-group comparisons.

**Table 3:** Known-group validity

	Overall (*n* = 419)	Nocturia < 2 episodes per night (*n* = 191)	Nocturia ≥ 2 episodes per night (*n* = 228)	Effect size^a^	*p*-value^b^
**Mean ICIQ-NQOL scores (SD)**					
Sleep/energy domain	4.4 (4.8)	2.8 (4.2)	5.8 (4.8)	0.66	< 0.001
Bother/concern domain	6.2 (4.9)	4.6 (4.6)	7.6 (4.6)	0.66	< 0.001
Total score	13.8 (11.0)	9.4 (10.1)	17.5 (10.3)	0.79	< 0.001

*Note *^a^Cohen’s *d* effect size

^b^Independent *t*-tests were used

*Abbreviations ICIQ-NQOL* International Consultation on Incontinence Questionnaire Nocturia Quality of Life Module, *SD* standard deviation

### Reliability

Regarding the sleep/energy and bother/concern domains, as well as the total scale, item-total correlations corrected for overlap exceeded 0.4 for all items, indicating strong associations with the respective domain and total scores of the ICIQ-NQOL. The Cronbach’s alpha coefficients for both domain scales and the overall scale were greater than 0.7, suggesting good internal consistency. These results are detailed in Table 4.

**Table 4:** Descriptive statistics, internal consistency of the ICIQ-NQOL

(*n* = 419)	Score = 4	Score = 3	Score = 2	Score = 1	Score = 0	Corrected Item-Total Correlation^a^	Corrected Item-Total Correlation^b^
	*n* (%)	*n* (%)	*n* (%)	*n* (%)	*n* (%)		
**Sleep/energy domain** (Cronbach’s alpha: 0.86)							
1. Concentration	11 (2.6)	29 (6.9)	65 (15.5)	65 (15.5)	249 (59.4)	0.82	0.73
2. Low energy	13 (3.1)	38 (9.1)	74 (17.7)	68 (16.2)	226 (53.9)	0.80	0.72
3. Sleep during the day	12 (2.9)	18 (4.3)	58 (13.8)	43 (10.3)	288 (68.7)	0.57	0.53
4. Productiveness	3 (0.7)	13 (3.1)	50 (11.9)	39 (9.3)	314 (74.9)	0.67	0.66
5. Physical activities	2 (0.5)	5 (1.2)	28 (6.7)	21 (5.0)	363 (86.6)	0.44	0.50
7. Inadequate sleep at night	35 (8.4)	64 (15.3)	92 (22.0)	84 (20.0)	144 (34.4)	0.62	0.69
**Bother/concern domain** (Cronbach’s alpha: 0.81)							
6. Fluid restriction	15 (3.6)	74 (17.7)	126 (30.1)	47 (11.2)	157 (37.5)	0.46	0.45
8. Disturbance of others	7 (1.7)	24 (5.7)	40 (9.5)	55 (13.1)	293 (69.9)	0.49	0.49
9. Preoccupation with waking at night	1 (0.2)	14 (3.3)	52 (12.4)	40 (9.5)	312 (74.5)	0.57	0.60
10. Worry over condition worsening	27 (6.4)	72 (17.2)	103 (24.6)	99 (23.6)	118 (28.2)	0.67	0.66
11. Worried over treatment options	14 (3.3)	39 (9.3)	63 (15.0)	66 (15.8)	237 (56.6)	0.61	0.59
12. Overall bother	32 (7.6)	56 (13.4)	111 (26.5)	97 (23.2)	123 (29.4)	0.65	0.75
13. Overall impact on everyday life: Mean (SD) 3.1 (2.5)							0.77
Cronbach’s alpha of the overall scale (item 1 to 13): 0.90							

*Note *^a^Correlated item-total correlation with the respective subscale. Item 13 does not belong to any subscale, so it was excluded from the analysis

^b^Correlated item-total correlation for all 13 items

*Abbreviation ICIQ-NQOL* International Consultation on Incontinence Questionnaire Nocturia Quality of Life Module, *SD* standard deviation

For the assessment of test-retest reliability, the follow-up questionnaire was completed by 51 participants two weeks after the baseline measurement. The ICCs were as follows: 0.69 for the sleep/energy domain scale, 0.58 for the bother/concern domain, and 0.65 for the overall scale. Further test-retest reliability was evaluated among 33 patients who reported no change in their nocturia symptoms. For this subgroup, the ICCs for both domain scales and the overall scale exceeded 0.7, indicating strong reliability over time. The test-retest reliability results are detailed in Table 5.

**Table 5:** Test-retest reliability

	Patients who have completed follow-up (*n* = 51)^a^	Patients with no change in nocturia symptoms (*n* = 33)^b^
**ICIQ-NQOL**	ICC (95% CI)	ICC (95% CI)
Sleep/energy domain	0.69 (0.51, 0.81)	0.73 (0.52, 0.85)
Bother/concern domain	0.58 (0.36, 0.74)	0.73 (0.51, 0.86)
Total score	0.65 (0.45, 0.78)	0.76 (0.57, 0.88)

*Note *^a^All patients who completed the 2-week follow-up assessment were included in the analysis (*n* = 51)

^b^A subset of patients who completed the 2-week follow-up assessment and who reported no change in their nocturia symptoms, as indicated by the Global Rating of Change scale, were included in the analysis (*n* = 33 out of 51)

*Abbreviations* CI: confidence interval; ICC: intraclass correlation coefficient; ICIQ-NQOL: International Consultation on Incontinence Questionnaire Nocturia Quality of Life Module

### Responsiveness

Among the 51 participants who completed the 2-week follow-up assessment, 17 reported improvement in their nocturia symptoms. This subgroup was used to evaluate the internal responsiveness of the ICIQ-NQOL to changes. The results of the paired *t*-test indicated no statistically significant changes in the ICIQ-NQOL sleep/energy domain, the bother/concern domain, and the total scores, with *p*-values greater than 0.05. However, the Cohen’s *d* effect sizes were 0.33 for the sleep/energy domain score and 0.42 for both the bother/concern domain and total scale scores. The results of the responsiveness are presented in Table 6.

**Table 6:** Responsiveness

(*n* = 17)^a^	Baseline assessment	2-week follow-up assessment	Effect size^b^	*p*-value^c^
**Mean ICIQ-NQOL scores (SD)**				
Sleep/energy domain	5.24 (4.84)	3.88 (3.20)	0.33	0.169
Bother/concern domain	8.00 (4.80)	6.24 (3.46)	0.42	0.185
Total score	16.71 (11.03)	12.88 (6.37)	0.42	0.145

*Note *^a^A subset of patients who completed the 2-week follow-up assessment and reported improvement in their nocturia symptoms, as indicated by the Global Rating of Change scale, were included in the analysis (*n* = 17 out of 51). Additionally, only 1 out of 51 patients reported deterioration. This participant was not included in the analysis

^b^Cohen’s *d* effect size

^c^Paired *t*-tests were used

*Abbreviations ICIQ-NQOL* International Consultation on Incontinence Questionnaire Nocturia Quality of Life Module, *SD* standard deviation

### Linear regression and moderated mediation analysis

Linear regression analysis revealed that the presence of nocturia (*β*: 6.53, *p* < 0.001), higher Global PSQI score (*β*: 1.46, *p* < 0.001), younger age (*β*: −0.28, *p* < 0.001), and male gender (*β*: 2.51, *p* = 0.011) were associated with poorer ICIQ-NQOL total score. The results of the linear regression analysis are presented in Table 7. The moderated mediation analysis supported the presence of conditional indirect effects, indicating that nocturia’s impact on HRQOL through sleep quality varies between sexes. The analysis showed that the indirect effect of nocturia on HRQOL via sleep quality was more pronounced in females than in males. This effect was significant across various domains, including the ICIQ-NQOL sleep/energy domain score (contrast: −1.35, 95% confidence interval [95% CI]: −2.35 to −0.47), the bother/concern domain score (contrast: −1.00, 95% CI: −1.80 to −0.34), and the overall total score (contrast: −2.85, 95% CI: −4.98 to −0.98). The result of the moderated mediation analysis is shown in Table 8.

**Table 7:** Linear regression to explore factors associated with ICIQ-NQOL scores

ICIQ-NQOL						
	Sleep/energy domain (*n* = 411)		Bother/concern domain (*n* = 411)		Total score (*n* = 411)	
	β (95%CI)	*p*-value	β (95%CI)	*p*-value	β (95%CI)	*p*-value
Nocturia *(Ref: no nocturia)*	2.11 (1.26, 2.96)	< 0.001	2.59 (1.68, 3.50)	< 0.001	6.53 (4.64, 8.42)	< 0.001
PSQI total score	0.68 (0.56, 0.81)	< 0.001	0.52 (0.39, 0.66)	< 0.001	1.46 (1.19, 1.74)	< 0.001
Age	−0.10 (−0.14, −0.05)	< 0.001	−0.12 (−0.17, −0.07)	< 0.001	−0.28 (−0.38, −0.17)	< 0.001
Male *(Ref: female)*	0.85 (−0.01, 1.72)	0.054	1.19 (0.27, 2.12)	0.012	2.51 (0.59, 4.44)	0.011
R^2^	0.33		0.26		0.36	

*Note *Nocturia was defined as experiencing two or more episodes per night

No other socio-demographic factors were included in the regression model except for age and sex

*Abbreviations*: *CI* confidence interval, *ICIQ-NQOL* International Consultation on Incontinence Questionnaire Nocturia Quality of Life Module, *SD* standard deviation

**Table 8:** Moderated mediation analysis

ICIQ-NQOL			
(*n* = 411)	Sleep/energy domain	Bother/concern domain	**Total score**
	Point estimate (95% CI)^a, b^	Point estimate (95% CI)^a, b^	Point estimate (95% CI)^a, b^
**Direct effect**	2.17 (1.32, 3.02)	2.67 (1.76, 3.59)	6.70 (4.80, 8.61)
**Indirect effect**			
Female	2.31 (1.43, 3.30)	1.71 (0.98, 2.60)	4.87 (2.99, 7.08)
Male	0.96 (0.49, 1.44)	0.71 (0.35, 1.15)	2.02 (1.04, 3.08)
*Contrast* ^c^	−1.35 (−2.35, −0.47)	−1.00 (−1.80, −0.34)	-2.85 (-4.98, -0.98)

*Note *^a^The number of bootstrap samples utilized to generate percentile bootstrap confidence intervals is 5000. A statistically significant effect at the 0.05 level (5% significance level) is indicated when the confidence interval does not include the value of 0. Age was controlled as a covariate in all models

^b^The interaction term between sex and nocturia significantly affected the PSQI total score, with a coefficient of −2.07 (95% CI: −3.33 to −0.80, p-value: 0.0014). Additionally, the test of the highest order unconditional interaction was statistically significant (*p*-value: 0.0014). The effect of nocturia on the PSQI total score was 3.54 (95% CI: 2.49 to 4.59, p-value: < 0.001) among females. The effect of nocturia on the PSQI total score was 1.47 (95% CI: 0.72 to 2.22, p-value: < 0.001) among males

^c^This note referred to the comparison of the conditional indirect effects of nocturia on health-related quality of life through sleep quality across sexes. The contrast is statistically significant, indicating that the indirect effect is more pronounced in females than in males

*Abbreviations CI* confidence interval, *ICIQ-NQOL* International Consultation on Incontinence Questionnaire Nocturia Quality of Life Module, *PSQI* Pittsburgh Sleep Quality Index, *SD* standard deviation

## Discussion

This study evaluated the content validity and psychometric properties of the ICIQ-NQOL within a primary care patient population in Hong Kong. The cognitive debriefing interviews, which included an equal gender distribution of patients with a mean age of 68 and varied educational backgrounds, along with healthcare professionals, provided robust CVI scores, confirming the clarity, relevance, and appropriateness of the questionnaire items. Evaluating content validity in female populations is crucial, especially considering that the ICIQ-NQOL was initially developed with a male-only cohort [14]. The relevance and appropriateness of the instrument’s content may not inherently translate to female patients, necessitating this rigorous validation process. Moreover, the assessment of the ICIQ-NQOL’s CVI among healthcare professionals is a pivotal step in ensuring the clinical pertinence of the scale. Their expert insights are invaluable in affirming that the questionnaire resonates with clinical realities and potentially enhances its utility in both research and practice.

The current study lends support to the concurrent and convergent validity of the ICIQ-NQOL. Considering that the modified IIQ-7 and IPSS serves as generic instruments for measuring LUTS and is not specific to nocturia, moderate correlations between these scales and the ICIQ-NQOL was anticipated. This outcome underscores the necessity for a more targeted instrument to accurately capture the distinctive effects of nocturia on HRQOL. Furthermore, we noted a moderate correlation between the ICIQ-NQOL and the PSQI, further corroborating the adverse impact of nocturia on sleep quality. This finding also echoes that from a validation study on male patients in Spain, which also reported a moderate correlation between the ICIQ-NQOL and the PSQI [37]. Significantly, when analyzing different component scores of the PSQI, the correlation strength between the ICIQ-NQOL and Component 1 (subjective sleep quality), as well as Component 7 (daytime dysfunction), was more pronounced than with other components. This suggests that nocturia markedly affects an individual’s perception of their sleep quality and their functioning during the daytime. This finding highlights the clinical importance of addressing nocturia as a condition that not only disrupts sleep but also has significant repercussions for daytime functioning and overall well-being.

The ICIQ-NQOL has proven to be a reliable patient-reported outcome measure, as indicated by satisfactory Cronbach’s alpha values, item-total correlations, and test-retest reliability. Contrastingly, a Spanish study reported a suboptimal Cronbach’s alpha of 0.655 for the bother/concern domain, suggesting limited internal consistency within that domain [37]. In our analysis, however, we observed a stronger Cronbach’s alpha for the same domain, which surpasses the results from the Spanish cohort. This discrepancy highlights the potential variability in the instrument’s performance across different populations and underscores the importance of context-specific validation to ensure accurate measurement of patient-reported outcomes.

This study was the first to evaluate the responsiveness of the ICIQ-NQOL. Our study found no statistically significant change between the baseline assessment and the 2-week follow-up. One possible explanation for this was the small sample size (*n* = 17) included in the analysis, which likely led to inadequate statistical power to detect a difference. Nonetheless, the Cohen’s *d* effect sizes were all larger than the recommended cutoff of 0.2, providing preliminary evidence to support the internal responsiveness of the ICIQ-NQOL. Our study findings were in line with those of a previous study that evaluated the internal responsiveness of the IIQ-7 among patients with LUTS [38]. That study found the standardized effect size to be 0.32 among patients with improved symptoms [38]. However, only one participant reported deterioration in symptoms, thus limiting our ability to assess the ICIQ-NQOL’s responsiveness to deterioration in this study. To provide more robust evidence regarding the responsiveness of the ICIQ-NQOL, further studies are needed with a larger sample size, including patients who experience both improvement and deterioration of symptoms, and longer follow-up periods. Additionally, external responsiveness, defined as the ability of the scale to detect a clinically important change over time with reference to an external criterion of health status, should also be further evaluated [30].

The moderated mediation analysis performed in this investigation offers critical insights into the intricate relationship among nocturia, sleep quality, and HRQOL, with notable distinctions based on sex. Although previous research has identified sleep quality as a mediator in the link between nocturia and HRQOL [39], the influence of sex as a moderating factor was not examined. Our study contributes to the literature by uncovering conditional indirect effects, indicating that the influence of nocturia on HRQOL through sleep quality exhibits variation among individuals. Notably, this effect is accentuated in females compared to males. There is a pressing need for additional research to dissect the reasons behind this disparity, examining both biological and psychosocial dimensions.

The finding that sleep quality mediates the relationship between nocturia and HRQOL supports the idea that interventions targeting both the reduction of nocturia symptoms and the enhancement of sleep quality are crucial for mitigating the negative impact of nocturia on HRQOL. However, the observed sex-specific variations highlight the critical need for gender-sensitive approaches in the clinical management of nocturia and its related symptoms. Developing strategies specifically designed to target nocturia and their subsequent effects on sleep may result in improved HRQOL outcomes, with a potentially greater benefit for female patients.

### Limitations

The study had several limitations that should be considered when interpreting the results. Firstly, the recruitment strategy was based on convenience sampling from public primary-care settings. This method excluded patients from private practices and secondary care facilities, potentially skewing the findings toward the demographic, socioeconomic, and health profiles typical of public healthcare system users. As a result, the study’s findings may not fully reflect the experiences of the broader population with nocturia, particularly those who seek care in private or other healthcare settings. Secondly, the sample size used to evaluate responsiveness was small. Thirdly, the psychometric properties of the measure were mainly evaluated among Cantonese speakers. To establish the measure’s reliability and validity across diverse language groups, it is essential to conduct further testing with Putonghua speakers and speakers of other Chinese dialects. This step is necessary to confirm that the measure performs consistently and accurately among different linguistic populations, ensuring its broad applicability in various Chinese-speaking communities. Finally, it is important to note that the cross-sectional nature of our analysis limits our ability to establish causality within the moderated mediation model.

### Implications

The findings from the current study suggest that the ICIQ-NQOL is a suitable instrument for evaluating HRQOL among patients with nocturia in primary care settings. In the realm of clinical practice, the tool’s demonstrated reliability and validity indicate its potential for inclusion as part of routine patient assessments. This could facilitate more structured and quantifiable evaluations of nocturia’s impact on patients, aiding in the personalization of treatment strategies and the assessment of treatment outcomes. In research contexts, the ICIQ-NQOL can serve as a standardized outcome measure for studies examining the epidemiology, burden, and treatment of nocturia. Moreover, the instrument’s application in multi-center and cross-cultural studies may enhance the generalizability of findings and support the development of evidence-based practices for managing nocturia’s impact on quality of life. The findings from our moderated mediation analysis suggest that adopting a gender-specific approach to evaluation and treatment may enhance the management of nocturia and improve its wider impact on HRQOL.

## Conclusions

In summary, the current study has established the ICIQ-NQOL as a reliable and valid tool for assessing HRQOL in primary care patients suffering from nocturia. The findings affirm the instrument’s utility in capturing the patient-reported impact of nocturia on daily living, supporting its integration into clinical practice for the targeted assessment and management of this condition. We also provided initial evidence regarding the responsiveness of the ICIQ-NQOL to changes. Besides, the moderated mediation analysis from our study highlights the complex and sex-specific relationships between nocturia, sleep quality, and HRQOL. The results suggest that the pathway through which nocturia affects HRQOL via sleep quality is more substantial in females, indicating a need for gender-tailored interventions.

## Data Availability

The datasets used and/or analysed during the current study are available from the corresponding author on reasonable request.
